# Thermal oxidation of nuclear graphite: A large scale waste treatment option

**DOI:** 10.1371/journal.pone.0182860

**Published:** 2017-08-09

**Authors:** Alex Theodosiou, Abbie N. Jones, Barry J. Marsden

**Affiliations:** The Nuclear Graphite Research Group (NGRG), School of Mechanical, Aeronautical and Civil Engineering, The University of Manchester, Manchester, United Kingdom; University of Liverpool, UNITED KINGDOM

## Abstract

This study has investigated the laboratory scale thermal oxidation of nuclear graphite, as a proof-of-concept for the treatment and decommissioning of reactor cores on a larger industrial scale. If showed to be effective, this technology could have promising international significance with a considerable impact on the nuclear waste management problem currently facing many countries worldwide. The use of thermal treatment of such graphite waste is seen as advantageous since it will decouple the need for an operational Geological Disposal Facility (GDF). Particulate samples of Magnox Reactor Pile Grade-A (PGA) graphite, were oxidised in both air and 60% O_2_, over the temperature range 400–1200°C. Oxidation rates were found to increase with temperature, with a particular rise between 700–800°C, suggesting a change in oxidation mechanism. A second increase in oxidation rate was observed between 1000–1200°C and was found to correspond to a large increase in the CO/CO_2_ ratio, as confirmed through gas analysis. Increasing the oxidant flow rate gave a linear increase in oxidation rate, up to a certain point, and maximum rates of 23.3 and 69.6 mg / min for air and 60% O_2_ respectively were achieved at a flow of 250 ml / min and temperature of 1000°C. These promising results show that large-scale thermal treatment could be a potential option for the decommissioning of graphite cores, although the design of the plant would need careful consideration in order to achieve optimum efficiency and throughput.

## Introduction

The UK currently has many graphite moderated reactors which have reached their end-of-life or will do so over the next ~10 years. Several of the older research reactors have long been shut down and, more recently, the last of the 26 Magnox reactors, Wylfa, has also been closed-down. The remaining 14 Advanced Gas-Cooled reactors (AGRs) currently in operation are now operating beyond their design lifetimes [1]. As a consequence of this, it is widely accepted by the nuclear community that the UK faces a significant decommissioning challenge over the next 10–100 years and a particular aspect of this task will be the disposal of a significant amount of irradiated graphite.

The Magnox and AGR’s contain large amounts of nuclear grade graphite, principally in the core, where it is primarily used as a neutron moderator, and also as a reflector [2–4]. It is estimated that, in the UK alone, that there is approximately 45,000 m^3^ of irradiated graphite residing within our reactors, equating to around 90,000 tonnes [5], with the global figure believed to be > 250,000 tonnes [6], all of which will require appropriate conditioning, treatment and disposal. Graphite also contains a variety of radionuclides, some long-lived such as ^14^C and ^36^Cl [6], and others considered short-lived such as, ^3^H and ^60^Co [7]; there are many other radionuclides present in the graphite, generally formed via the neutron induced activation of metallic impurities inherent to the origin and manufacture of the material [7].

At present, the UK Nuclear Decommissioning Authority (NDA) baseline strategy is to suspend the abstraction of graphite cores, during a period of “care and maintenance” typically in the order of > 85 years, after which the graphite will be removed and conditioned before being disposed of in a future geological disposal facility (GDF) [8]. This approach, however, has several drawbacks and is likely to be both time consuming and expensive, particularly since the graphite waste is believed to be the single largest volume waste stream in the UK inventory [9].

Another possible approach for graphite could be large scale gasification; under optimum conditions, graphite is known to readily oxidise to the gas phase i.e. gasification, the topic of which has provided a wealth of literature [10–12]. These previous authors have monitored the gasification process by measuring the oxidation-induced mass loss of samples *in situ*, through thermal gravimetric analysis (TGA); however this method offers little insight into the gaseous species being evolved as a result of oxidation. This study employs an alternative approach using quantitative gas analysis (QGA) to directly monitor the off-gasses produced through oxidation along with their production rates in real-time; hence providing an improved understanding of the oxidation mechanisms involved.

Much of the more recent work looking at graphite oxidation has focused on relatively new graphite grades aimed at high-temperature gas reactors (HTGRs) [13,14]. In particular, grades such as IG-110, IG-430, NBG-18 and NBG-25 are of interest [15,16] with primary focus on operational safety during steam [14,17] or air [18,19] ingress faults.

Whilst this work is important for future reactor designs and power generation, this study focuses on graphite oxidation from a waste treatment standpoint, the results of which could be used to inform optimum decommissioning strategies for the reactors that have reached end of life. As a consequence, this paper is based on the laboratory-scale gasification of nuclear grade graphite grades that were extensively used in the UK Magnox reactors as a neutron moderator; this graphite is known as Pile Grade-A (PGA). PGA graphite bricks were used to construct the moderator cores for the Magnox fleet and therefore forms the majority of the UK graphite waste inventory currently ready for disposal. Further information on the origin and nature of PGA graphite can be found elsewhere [20–22]. This work aims to be a proof-of-concept for possible pilot scale gasification of reactor cores leading to accelerated decommissioning of the UK Magnox and older research reactor fleet; If successful, a large scale thermal treatment plant could be potentially deployed to a variety of graphite-containing reactor sites worldwide. Such treatment would lead to a very large volume reduction in irradiated graphite and provide an efficient, time reducing and cost-effective decommissioning option which may decouple the need to store graphite in a GDF. However, the applicability of this method would depend largely on the available options to deal with the large amounts of gaseous ^14^C being produced, such as potential aerial discharge (according to local regulations) or carbon-capture followed by liquefaction and storage.

## Materials and methods

### Graphite samples

Graphite particulate was generated by crushing graphite blocks using a jaw crusher and separating the resulting debris into various sizes using a vibrating sieve shaker. The starting material was a large moderator brick of Bradwell (UK) PGA which is an un-irradiated (virgin) replica of the moderator bricks currently present within the closed down Bradwell reactor. This block was machined into cubes of dimensions 20 x 20 x 20 mm, which were subsequently passed through the jaw crusher. A particle size range of 2.0–6.0 mm was deliberately chosen to best resemble the size of particulate likely to be generated by the graphite removal process and one that could easily transfer to an industrial feeding system if required.

### Apparatus and methodology

**Fig 1:**
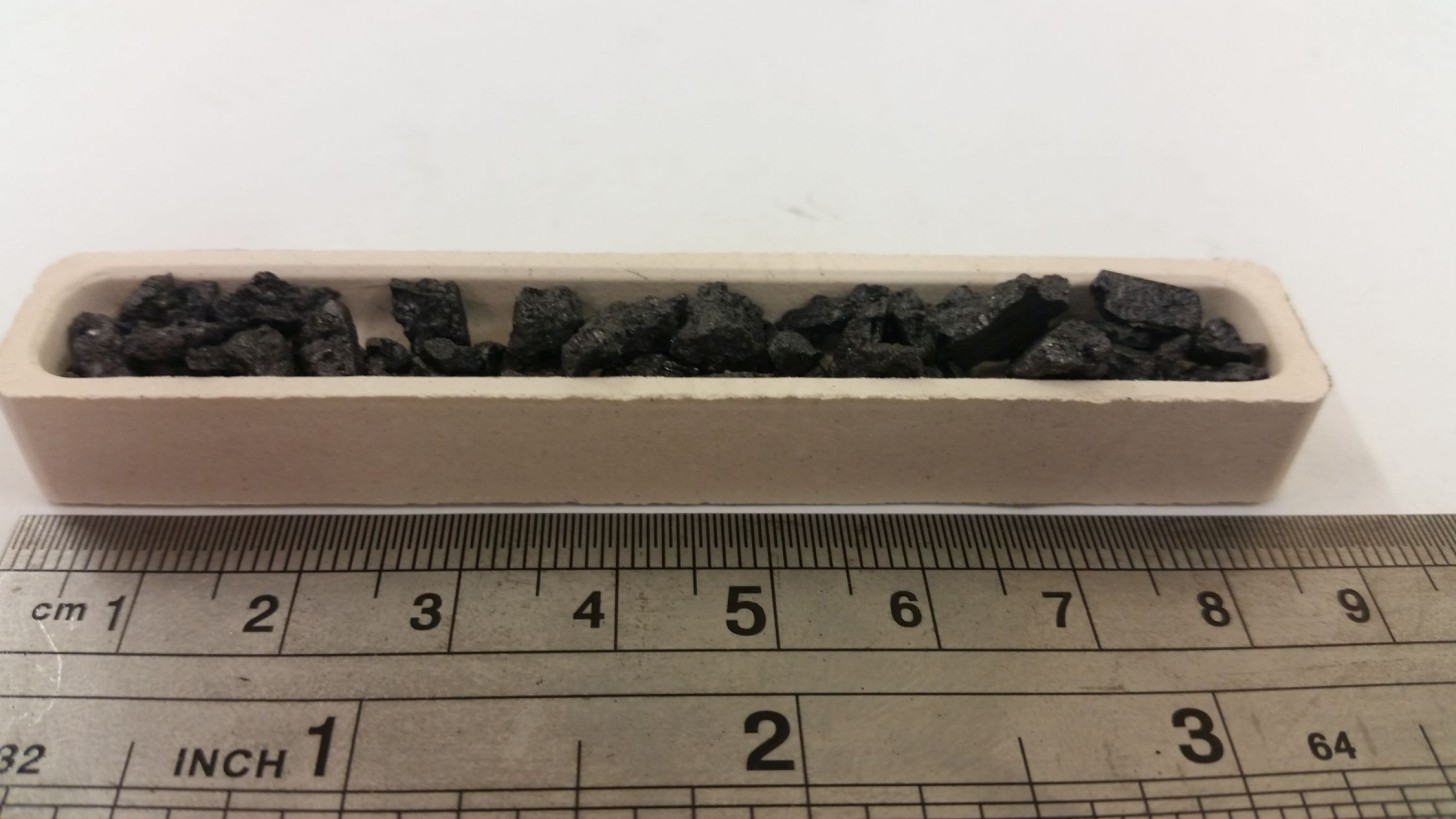
A ceramic combustion boat filled with ~ 3.0 g PGA particulate of size 2.0–6.0 mm.

Particulate, of approximately 2.0–6.0 mm in size, was weighed out into 3.0 g (± 0.01 g) samples which were placed into a ceramic combustion boat (c.f. Fig 1) before being inserted into a horizontal tube furnace (T_max_ = 1200°C). The inlet to the furnace was connected to high purity oxidant gas bottles via a digital mass flow controller and the flow rate was set to a fixed value of 100 ml/min (± 1 ml / min), unless stated otherwise. The oxidant gases used in this study are dry air and 60% O_2_ in argon (N6 purity). Once the sample was loaded, the furnace was sealed and purged with Ar to create an inert atmosphere. This process inhibits any oxidation taking place before the desired temperature is reached. Once at temperature, the Ar was switched off and the oxidant was switched on simultaneously; this initiates the start of the experiment. Each experiment was 60 mins long in order to achieve a reasonable weight loss. Oxidation is terminated by closing the supply of oxidant gas and re-introducing Ar into the system. This ensures that no further oxidation occurs during the cooling down process. Once cooled the sample was re-weighed in order to obtain the oxidation-induced weight loss.

The furnace outlet was connected to a quantitative gas analyser (QGA, Hiden Analytical®) equipped with a backing pump to draw the gas into the detector through a ‘hot-stage’ assembly. The hot-stage was kept at a temperature of ~ 200°C to help to minimise any condensation of gaseous species before they reach the detector. Real-time analysis of CO and CO_2_ release was used to obtain the graphite oxidation rate throughout the course of the experiment. The results were checked against the actual weight loss measurements and found to be in excellent agreement.

**Fig 2:**
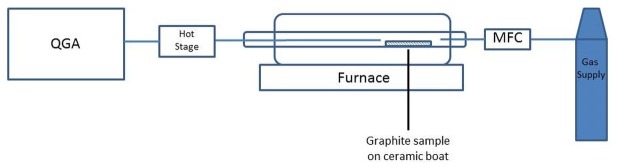
A schematic of the thermal treatment apparatus used.

A schematic of the laboratory setup is shown in Fig 2.

## Background

### Graphite oxidation reactions

The oxidation of graphite is complex, consisting of a series of chemical and physical steps determined by a variety of thermodynamic and kinetic factors [23]. Some of the better known chemical reactions between carbon and oxygen are outlined below, although the precise nature of the mechanisms and pathways involved are still a topic of debate.

Under certain conditions CO may be the primary product with the reaction proceeding as:

$$C_{g}+\frac{1}{2}O_{2}\to \mathrm{CO}$$ (Eq 1)

Where C_g_ refers to carbon in its solid state as graphite.

Alternatively molecular oxygen may adsorb to the graphite surface and rearrange to give CO via the oxidation of a neighbouring atom [24]:

$$C_{g}+C_{g}(O_{2})\to 2CO$$ (Eq 2)

However, CO_2_ is also formed through graphite oxidation and the mechanism is perhaps even more complex, with several proposed pathways available in the literature [24–26]. CO_2_ production can either occur through further reactions of C_g_-O:

$$C_{g}O+\frac{1}{2}O_{2}\to {\mathrm{CO}}_{2}$$ (Eq 3)

$$2C_{g}O\to CO_{2}+C_{g}$$ (Eq 4)

$$C_{g}O+CO\to C_{g}+CO_{2}$$ (Eq 5)

$$2C_{g}O+CO\to CO_{2}+C_{g}O+C_{g}$$ (Eq 6)

Or, straight through as a single step process [27]:

$$C_{g}{+O}_{2}\to {\mathrm{CO}}_{2}$$ (Eq 7)

Numerous authors have suggested the primary mechanism, responsible for graphite oxidation, to be multi-step, beginning with the arrival of the oxidant to the graphite surface [28–30]. In the case of molecular oxygen, two intermediate configurations are possible depending on how the O_2_ molecule adsorbs to the surface. It can either physisorb onto the surface and form a weak bond with the graphite, or it can chemisorb to the underlying C-C bonds forming new C-O bonds; both options appear to be dependent on available energy within the system and any defects/vacancies present on the graphite surface [31].

Gasification of the graphite then proceeds through the breaking of C-C bonds, via the decomposition of intermediate oxygen complexes. El Genk et al [32] have proposed a variety of possible oxygen-containing organic complexes which may be formed which, at elevated temperatures, may thermally desorb CO into the gaseous phase.

Regarding CO_2_ formation, El-Genk also proposed that incoming oxygen molecule may react with a reactive carbon atom that has had its C-C bond weakened due to prior chemisorption of oxygen. Alternatively, CO_2_ may form through reactions with CO molecules in a secondary oxidation process, either reacting with more oxidant in a gas phase reaction or by reacting with nearby physi/chemisorbed oxygen to produce CO_2_ [33] via Eq (5).

### Thermodynamics of graphite oxidation

**Fig 3:**
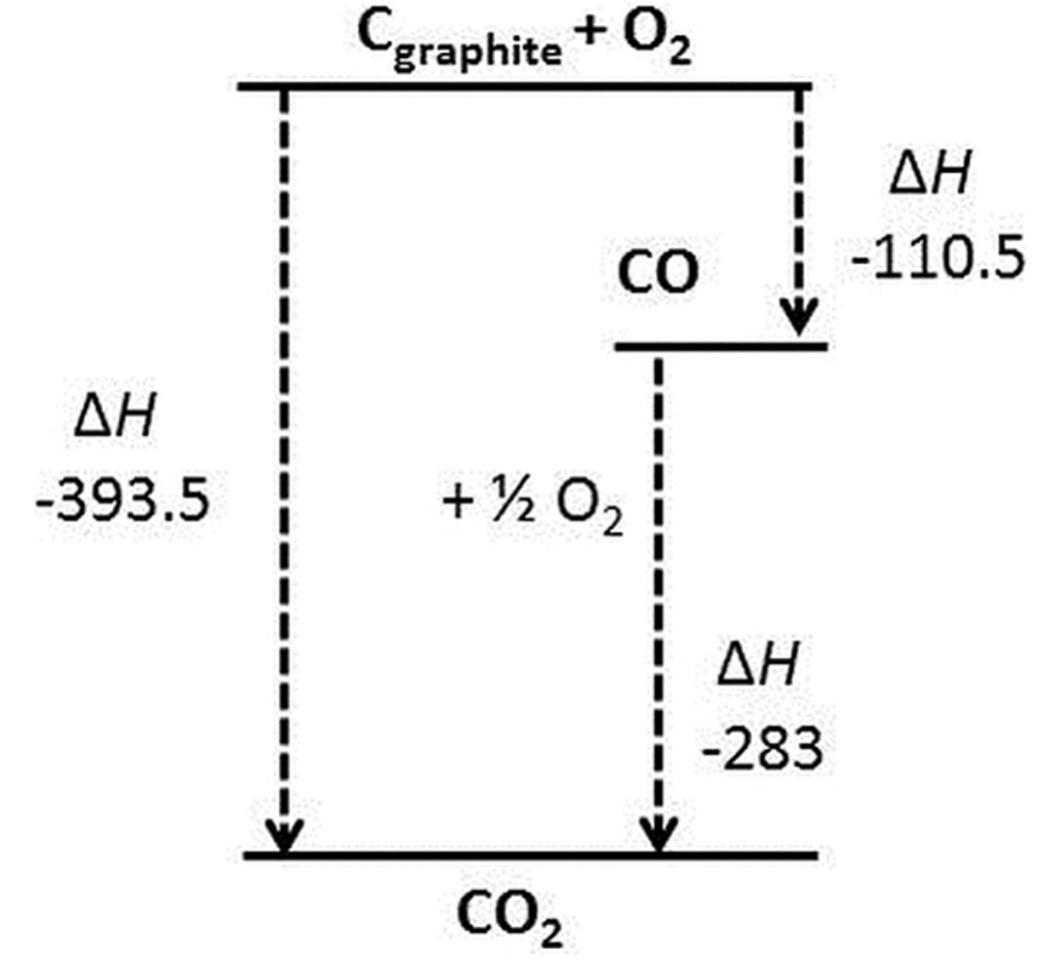
An energy level diagram of the oxidation of graphite to CO_2_. Δ*H* is given in kJ/mol.

An energy level diagram of the oxidation of graphite to CO_2_ is shown in Fig 3, where the overall change in enthalpy of the system i.e. the change in energy at constant pressure, Δ*H*, can be seen to be unaffected by any intermediate stages, as defined by Hess’ law. However, it can be seen that the initial step of the 2-step pathway i.e. the production of CO releases less heat into the system than straight conversion to CO_2_. On a large scale industrial process this difference may be of importance to the plant design and operation.

Using Δ*H°*, the Gibbs free energy at standard temperature and pressure (STP = 298.15 K and 1 bar), Δ*G°*, can be calculated using Eq (8), which takes into account the change in entropy of the system, Δ*S°* i.e. S° (products)—S°(reactants).

$$\Delta G^{o}=\Delta H^{o}-T([S^{o}({\mathrm{CO}}_{2})]-[S^{o}(C)-S^{o}(O_{2})])$$ (Eq 8)

Inserting literature values for *S*° of 5.75, 205.14 and 213.74 JK^-1^/mol for C_g_, CO and CO_2_ respectively at STP [34], gives Δ*G*° = -394.4 kJ/mol. This calculated Gibbs energy value indicates that graphite oxidation is thermodynamically very favourable since a reaction may occur spontaneously, under constant conditions, when Δ*G*° < 0. However, since graphite oxidation is not measurable at room temperature then the reaction is assumed to be occurring at a negligible rate, that is, kinetically unfavourable.

### Graphite oxidation rate

Graphite oxidation is known to consist of several kinetic regimes, sometimes also referred to as ‘oxidation modes’, beginning at temperatures > 400°C. There is a wealth of literature in this area [35–37] and therefore only a short explanation will be recapped for scientific completeness. The modes are classified as follows:

Mode A: A chemical rate regime (low temperature)
Mode B: An in-pore diffusion controlled regime (intermediate temperature)
Mode C: A boundary-layer controlled regime (higher temperatures)

**Fig 4:**
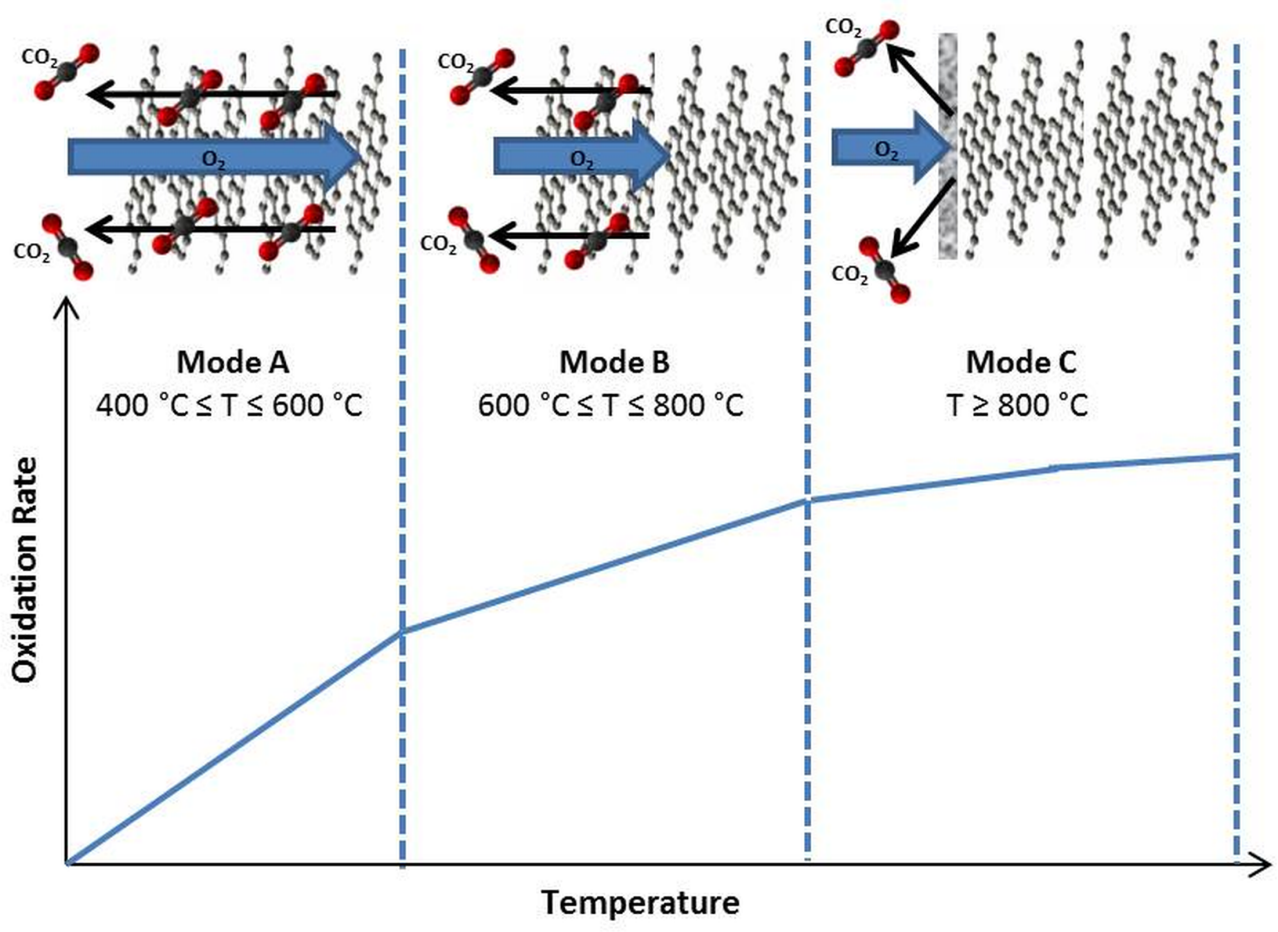
The various kinetic regimes thought to be involved in graphite oxidation. Artwork inspired by Clark et al [38]. The temperature ranges shown are an approximation and can vary depending on many factors.

Each mode occurs within a specific temperature range, however there is some dispute in the literature as to what these temperature ranges are [37] mainly due to experimental differences involved in much of the research and the complexity of the graphite oxidation itself. An interpretation of the oxidation mechanisms occurring in the various modes is shown in Fig 4. The differing oxidation modes, and the processes involved, can be used to explain the change in oxidation rate with temperature.

In mode A, oxidation occurs within the bulk of the graphite as the oxygen molecules are allowed to permeate through the surface and react within the active pore volume. Oxygen is able to penetrate relatively deep into the graphite pore volume before it is consumed by reacting to form CO or CO_2_. Oxidation in this mode essentially occurs from the inside out, eroding the internal pores structure of each particulate before eventually the changes in surface area cause the oxidation to take over the whole sample leading to complete gasification. Within this regime the rate limiting factor is the chemical reaction itself since the time taken for the reacting species to reach the active sites is relatively fast [38].

In mode B, oxygen molecules are not able to travel as deep into the graphite pore structure, becoming hindered by a counter-current of CO and CO_2_ emanating from the internal porosity. Within this mode the oxidation rate is said to be diffusion controlled. The concentration of oxidant within the pore structure and hence the erosion of the internal porosity decreases with penetration depth and, as the temperature increases, the majority of the reactions start to take place at the outer surface.

In mode C, oxidation occurs only on the outer surface, leading to dramatic and rapid erosion of the external surface of the particulate, which can quickly result in full gasification. The rate within this regime is limited by the diffusion of oxidant through the boundary layer which is also competing with counter diffusion of CO and CO_2_ from the bulk.

## Results and discussion

### Effect of temperature

**Fig 5:**
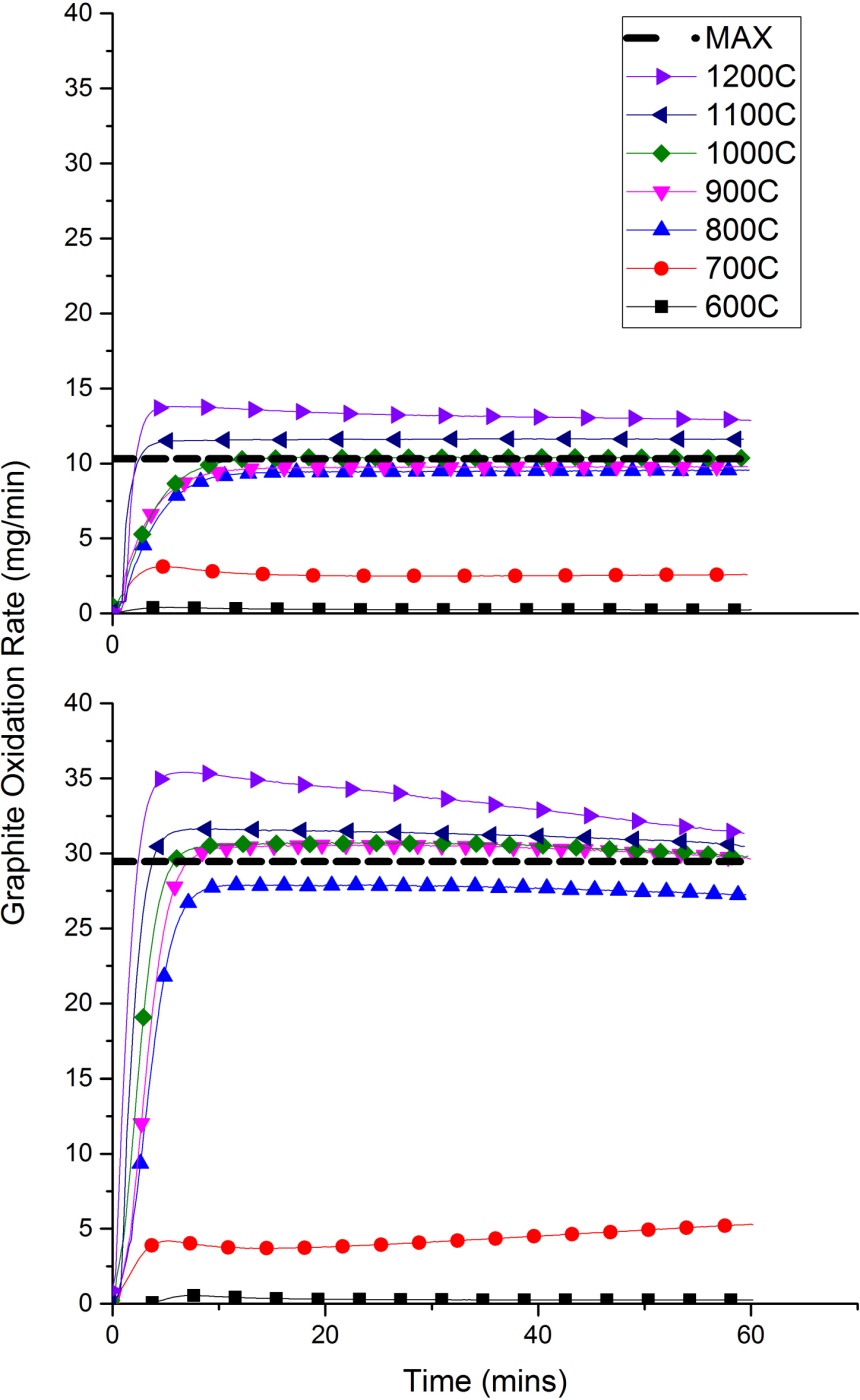
The effect of temperature on graphite oxidation rate in air (top) and 60% O_2_ (bottom).

Fig 5 shows the present author’s results for the oxidation behaviour of PGA, over time, at temperatures of 600–1200°C in air and 60% O_2_. Experiments were also carried out at 400 and 500°C but these are omitted as the oxidation rates were too low to measure confidently and therefore considered to be negligible. The line labelled as ‘MAX’ refers to the maximum oxidation rate that could be achieved if the reaction proceeded to produce CO_2_ in a single step i.e. Eq (6). These “MAX” values have been calculated as 10.31 and 29.45 mg/min for air and 60% O_2_ respectively.

The data shown in Fig 5 combines contributions from both CO and CO_2_ to give an overall oxidation rate and it is seen that the same general trends are observed for both air and 60% O_2_, which is primarily an increase in oxidation rate with increasing temperature. In each case, the oxidation profile displays an initial rapid increase in oxidation rate, leading up to a steady-state plateau. For 60% O_2_, higher temperature experiments exhibit a drop in oxidation rate towards the end of the 60 mins. This decline in oxidation rate is due to the total remaining mass decreasing as the sample gasifies, and therefore there is less graphite available to oxidise. This effect is not observed at 600⁰C since the oxidation rate is much slower and a large percentage of the original graphite mass is still remaining. Similar trends have been recorded in the literature and attributed to surface area effects [39]. This same effect is not observed in air until 1200°C since the oxidation is much slower and so the total graphite mass is relatively unchanged at the end of the experiment.

A large increase in oxidation rate is observed between 700–800°C in both experiments, with a three times increase in air and almost a five times increase in the case of 60% O_2_. This data suggests that, at 700°C, oxidation is occurring viamode B and that the onset of mode C occurs at some temperature between 700 and 800°C. The observed dramatic jump in oxidation rate implies that the transition between modes B and C is particularly sudden and temperature sensitive. Interestingly, the increase in rate between 600–700°C is relatively small and may indicate an extension of the chemical controlled regime to temperatures closer to 700°C, rather than the widely reported 600°C [32,35,40] suggesting that, with these specific conditions, the temperature range for mode B is quite narrow, approximately between 700–800°C. These differences in observed transitions are a result of many influencing factors such as microstructure, sample geometry, density and impurity content, which can vary widely between researchers, experimental set-up and graphite grade[41].

In contrast, the increase in oxidation rate between 800–1000°C is comparatively small, especially so from 900–1000°C. Here the reaction appears to be in mode C, proceeding rapidly with almost all of the oxidant being consumed at the graphite surface. Increasing the temperature has little effect on the oxidation rate in this regime and the reaction can be considered to be in a steady-state. Interestingly, at these high temperatures (T ≈ 1000°C) the observed oxidation rates start to exceed the ‘maximum line’ corresponding to the oxidation rate expected by assuming only CO_2_ production i.e. at least some proportion of the graphite is oxidising to form CO, rather than CO_2_.

**Fig 6:**
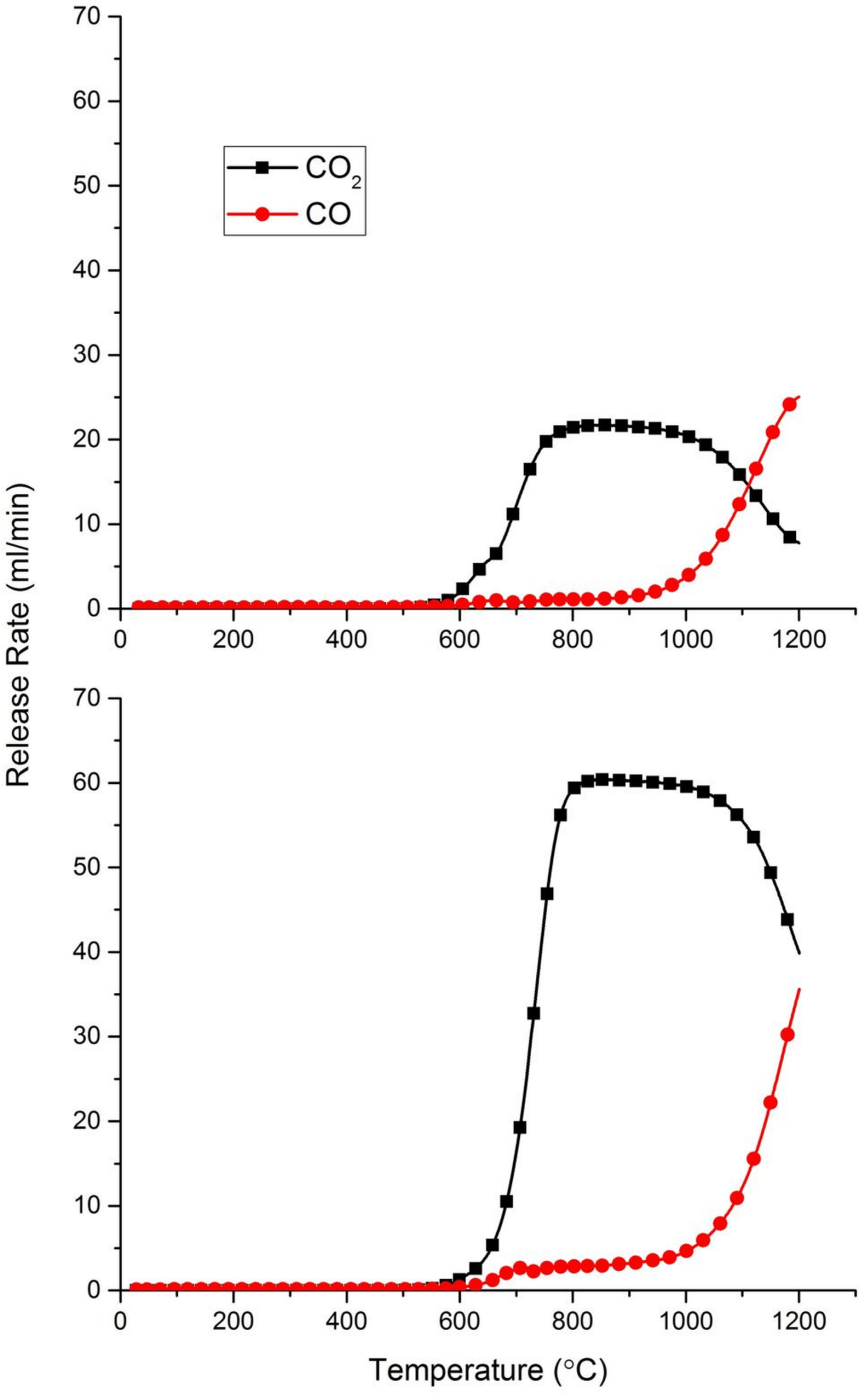
The effect of temperature on the CO/CO_2_ production ratio in air (top) and 60% O_2_ (bottom).

Between 1000–1200°C, the oxidation rates begin to increase further, particularly so at 1200°C, reaching 13.8 and 35.4 mg/min for air and 60% O_2_ respectively. The reason for this increase can be seen in Fig 6, which shows the CO and CO_2_ release rates with increasing temperature. A significant increase in the amount of CO being produced at temperatures ≥ 1000°C is observed, reaching a maximum at 1200°C. In the case of air, CO/CO_2_ > 1 at ~1150°C, reaching a maximum of 3.2 at 1200°C. Similar observations have been made previously looking at char oxidation with an interesting overview carried out by Shaddix et al [42]. In agreement with this present study, experiments carried out by Takahashi et al [43] on nuclear graphite demonstrated a rapid increase in CO/CO_2_ at around 1000°C, and postulated that the reason for the results was an increase in the endothermic Boudouard reaction (C +CO_2_), which is favoured at higher temperatures [44]. Du et al [45] suggested that the general increase in CO/CO_2_ with temperature is due to the higher activation energy for CO production compared to that of CO_2_. This paper, and others [46,47], also found that CO/CO_2_ decreases with increasing oxygen concentration, which helps to explain why in Fig 6 the relative increase is higher in air than that in 60% O_2_.

**Fig 7:**
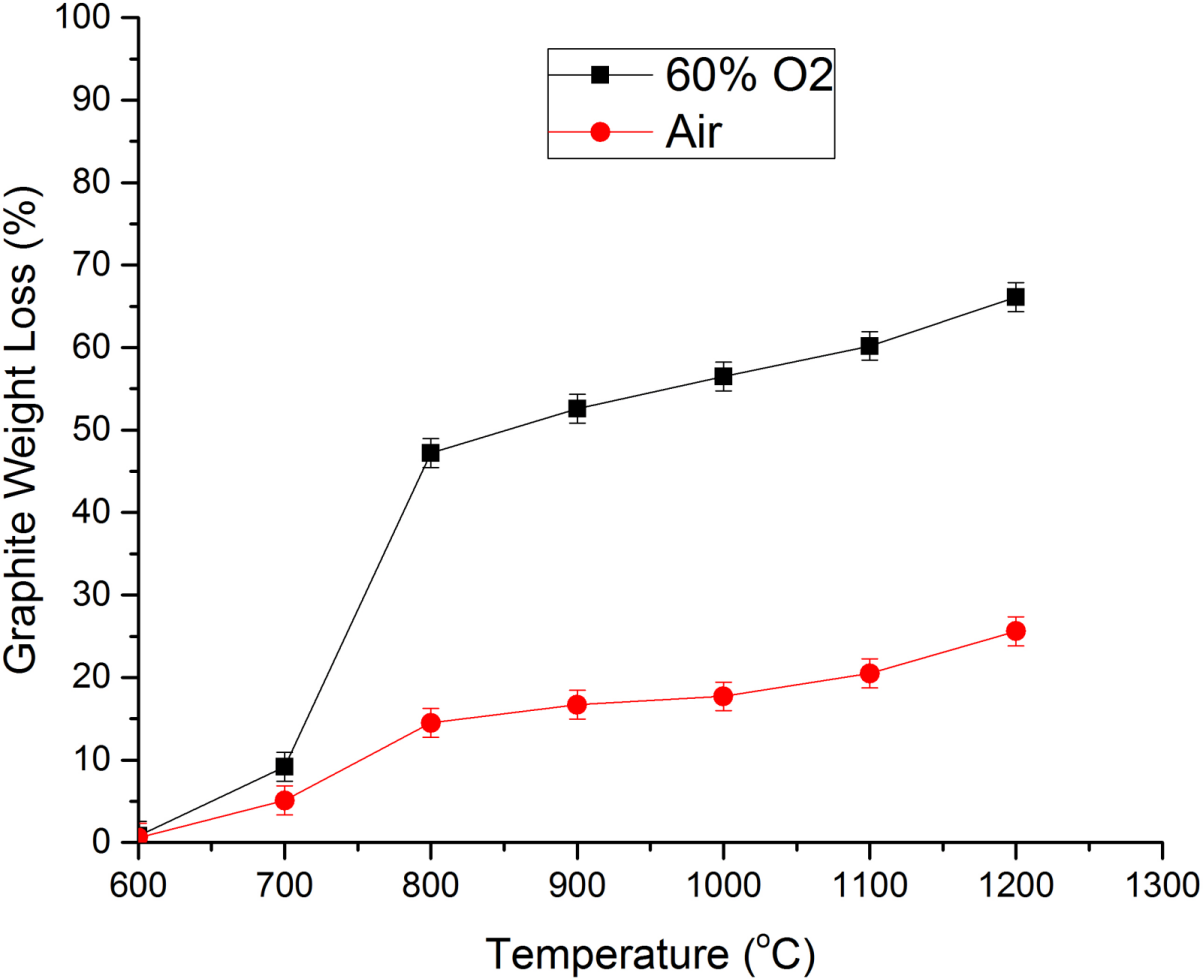
Comparison of weight loss against temperature in air and 60% O_2_.

A measurable effect of this CO/CO_2_ increase is the corresponding increase in oxidation induced weight loss, since each oxygen molecule is able to remove 2 carbon atoms for CO formation, compared to a single carbon atom for CO_2_ formation. Table 1 contains the measured weight loss data for the performed experiments, which are plotted out in Fig 7.

**Table 1:** Measured weight loss after 1 hour oxidation at various temperatures for an initial graphite mass of ~ 3.0 g (± 0.01 g). The flow rate was fixed at 100 ml/min.

	Air (21% O_2_)		60% O_2_	
Temperature (°C)	Weight Loss (g)	Weight Loss (%)	Weight Loss (g)	Weight Loss (%)
600	0.0189	0.62	0.0236	0.8
700	0.1532	5.1	0.2758	9.2
800	0.4338	14.5	1.414	47.2
900	0.5029	16.7	1.5766	52.6
1000	0.5296	17.7	1.6954	56.5
1100	0.6176	20.5	1.811	60.2
1200	0.7677	25.6	1.9824	66.1

At lower temperatures, T ≤ 600°C, the measured weight loss is relatively unaffected by the increase in oxygen concentration; this is due to the relatively low number of oxygen molecules with energy greater than or equal to the activation energy, *E*_*a*_ at this temperature. Within this temperature regime the reaction can be said to be close to zero-order with respect to O_2_. At 700°C, the gap widens slightly, but not as much as expected if the oxidation mechanism had fully changed. This corroborates the suggestion that at this temperature the majority of the reactions are still occurring via the chemical controlled regime i.e. mode A. As discussed, within this mode the rate limiting step is the transport of the oxidising species through the bulk to the open pore volume. It is then feasible that increasing the number of oxidising species may not necessarily lead to a proportional increase in oxidation rate since there would be enhanced competition to the active sites and the potential for oxygen molecules to block each other within the micropores. Also, increasing the oxygen concentration will lead to a larger amount of surface oxides, therefore reducing the number of free active sites available to react [48].

As the temperature increases to T ≥ 800°C, and the reaction starts to enter mode C, the effect of oxygen concentration becomes more pronounced with the difference in weight loss almost becoming proportional to the increase in oxygen concentration i.e. the reaction is approaching first order. The increase in temperature means that a larger proportion of the molecules now have E > *E*_*a*_ and are therefore able to react successfully, as defined by the Maxwell-Boltzmann distribution curve.

Interestingly, at 1200°C, the effect of the increase in oxygen concentration is slightly diminished due to the preferential CO production observed at lower concentrations [45], thus leading to a relatively larger increase in weight loss under these conditions. This is of importance for industrial up-scaling of the method, where air may be the preferred oxidant, suggesting that proportionately higher oxidation rates could be achieved by increasing the temperature to ≥ 1200°C. This would have a significant effect on throughput and allow for improved control over the amount of heat generated within the system, as shown in Fig 3.

### Kinetics of graphite oxidation

**Fig 8:**
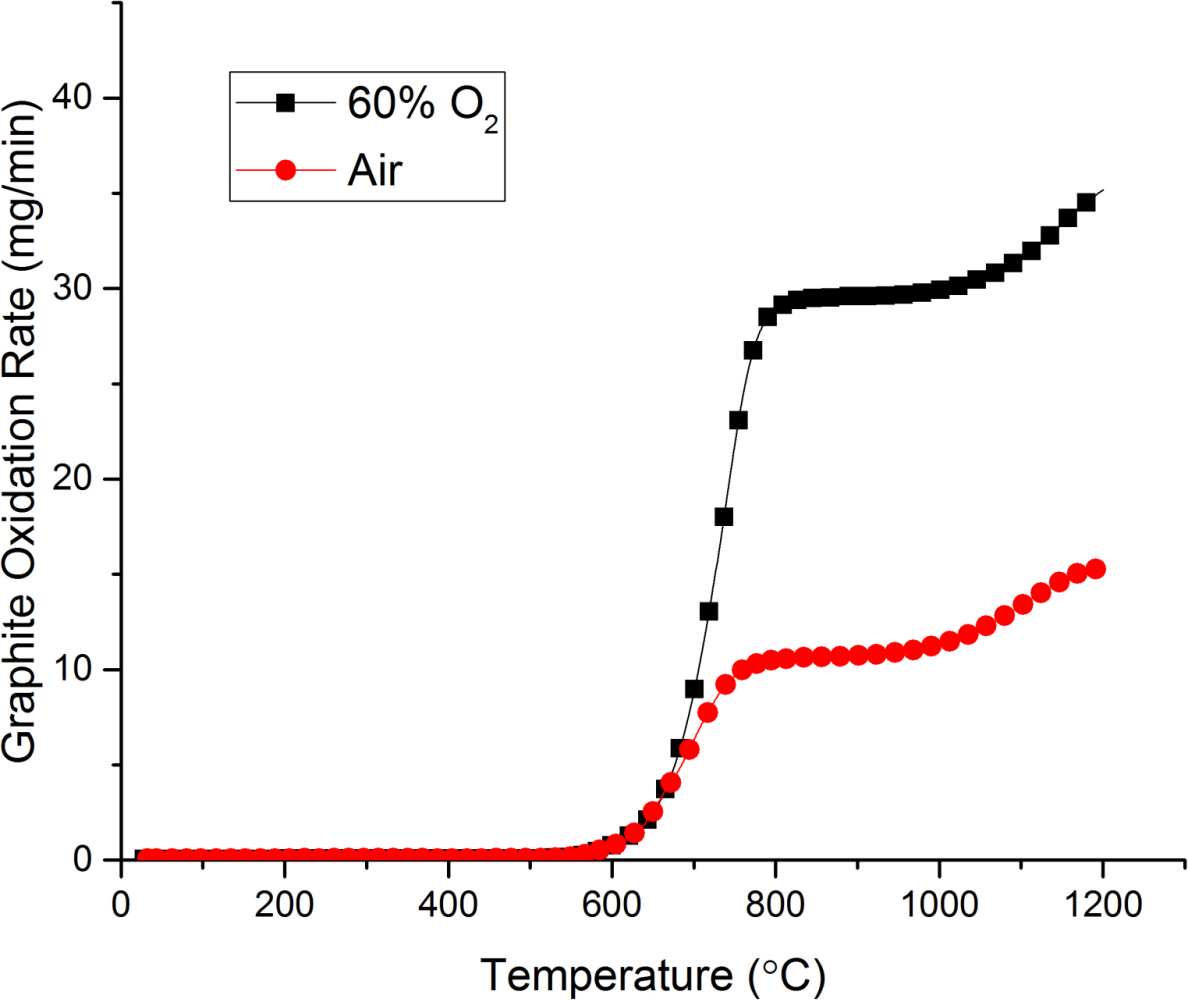
The effect of increasing temperature on the graphite oxidation rate for both air and 60% O_2_.

In this experiment, the temperature dependence of graphite oxidation was studied over a wider temperature range of 30–1200°C. The heating rate was set to 5°C / min in order to ensure a large amount of data was collected. The oxidant flow rate was again fixed at 100 ml / min. The results are shown in Fig 8.

**Fig 9:**
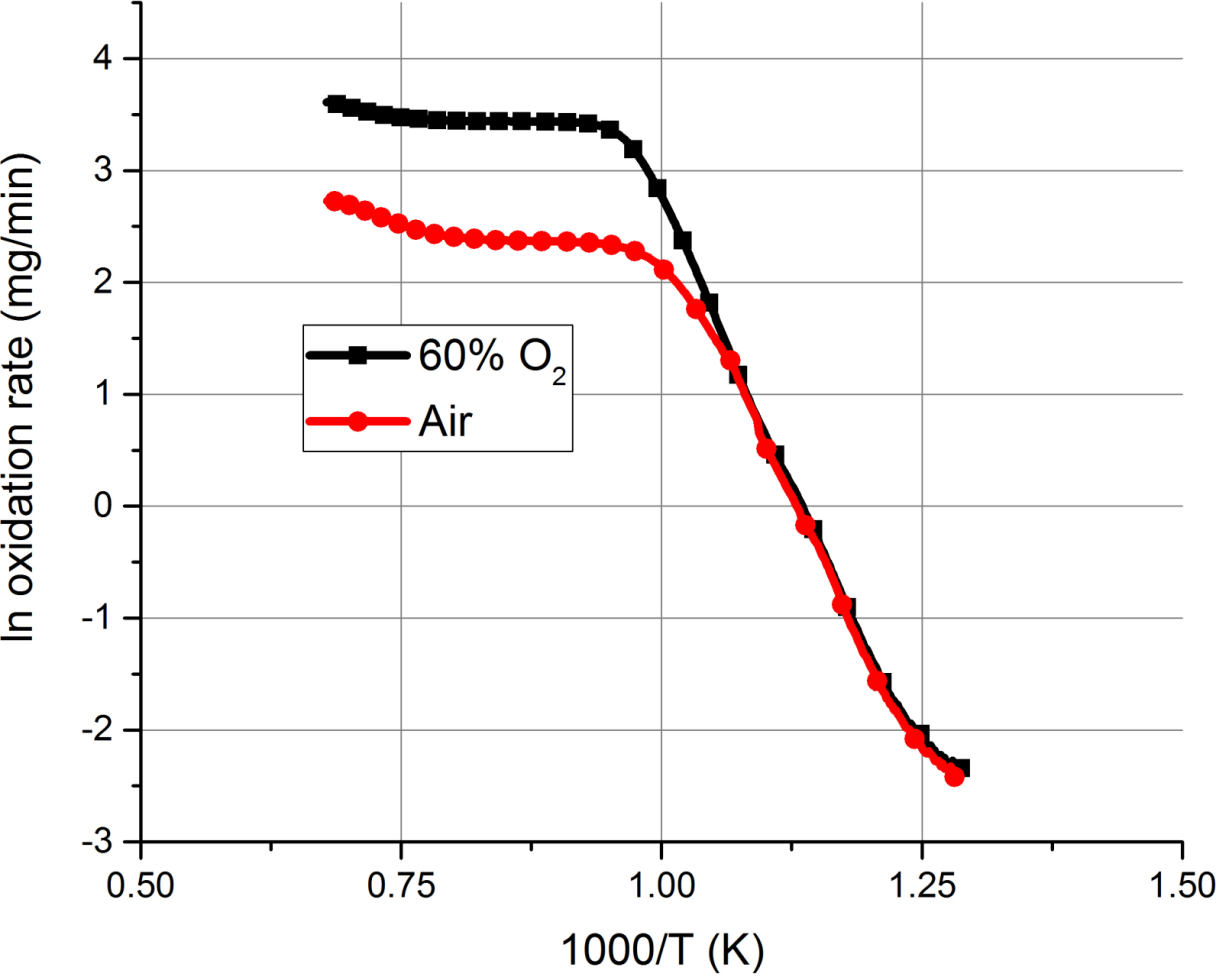
Arrhenius plot showing the temperature dependence of graphite oxidation rates from 500–1200°C. Data from T < 500°C is omitted due to negligible oxidation rates.

The observed relationship between temperature and oxidation rate has been discussed previously. This data may be re-plotted in the Arrhenius form (Fig 9) to provide the activation energy of the various kinetic regimes [39] where the gradient is equal to *-E*_*a*_*/R* and the intercept is equal to the pre-exponential factor, ln *A*, as described by Eq 9.

$$\ln\;k=-\frac{E_{a}}{R}.\frac{1}{T}+\ln\;A$$ (Eq 9)

Where *k* is the oxidation rate, *T* is the temperature in kelvin and R is the universal gas constant.

The data in Table 2 was obtained by fitting a linear best fit line to the various temperature regimes stated. However, as discussed previously, the transition temperature between these regimes can involve significant overlap, hence this empirical approach should be taken as a guideline only [49]. Here the regimes chosen were taken from analysis of the oxidation experiments and optimisation of the R^2^ value given to the line of best-fit. Due to the very distinct and considerable increase in oxidation rate observed at T ≥ 1000°C, mode C has been split into two parts, of low CO and high CO, spanning the temperature ranges of 800–1000°C and 1000–1200°C, respectively.

**Table 2:** The data derived from fitting a linear line of best-fit over the various temperature regimes in the Arrhenius plot. Values of *E*_*a*_ are in kJ/mol.

	Air				60% O_2_			
Temp Range (°C)	ln A	Slope	E_a_	R^2^	ln A	Slope	E_a_	R^2^
500–650	19.98	-17.72	147.33	0.9878	23.31	-20.09	167.04	0.9948
650–800	10.81	-8.83	73.38	0.9056	16.74	-14.00	116.37	0.9542
800–1000	2.81	-0.51	4.24	0.866	3.55	-0.14	1.14	0.8404
1000–1200	4.93	-3.21	26.73	0.9932	4.68	-1.59	13.24	0.9536

Firstly, for oxidation in air, the calculated values of the activation energies agreed very well to those reported in the literature, particularly with the work carried out by Luo et al[39]. In Mode B, the activation energy is halved to 73.4 KJ/mol. This again is consistent with the literature and is seen as an indicator of mode B kinetics [39,50,51].

At higher temperatures, during mode C, the measured *E*_*a*_ is very low, explaining the high oxidation rate observed since almost all the oxygen molecules will have E > *E*_*a*_. However, at T > 1000°C, *E*_*a*_ is seen to increase again reaching a value of 26.7 kJ / mol over the range 1000–1200°C. This increase is attributed to the higher CO production occurring within this temperature range and is likely caused by the greater specific energy of desorption of a CO molecule relative to a CO_2_ molecule [52].

Results in 60% O_2_ are interesting and follow the same general pattern as in air, however the values of *E*_*a*_ are somewhat different. For T ≤ 800°C the *E*_*a*_ values are slightly higher than those observed for air over the same temperature range but lower from 800–1200°C. Such differences can be expected, since *E*_*a*_ is known to be sensitive to many factors, and a wide range of values have been reported in the literature. The presence of metallic impurities within the graphite is one such factor, which can catalyse oxidation by lowering the activation energy [53] and although the graphite particulate used was generated from the same block, impurity content could be a source of variation between samples. Additionally, Li and Brown [54] found the CO/CO_2_ ratio to also effect the activation energy, since this is dependent on oxygen concentration and therefore could explain the differences observed.

### Effects of flow rate

The oxidation rate is ultimately limited by the number of available oxidant molecules, in this case O_2_, that are available to react per unit time; hence for a known flow rate, the maximum oxidation rate can be calculated. Table 3 shows the maximum oxidation rate available for a range of different flow rates based on the carbon-oxygen reaction proceeding via Eq (7).

**Table 3:** The effect of O_2_ concentration and flow rate on the maximum graphite oxidation rate. The figures assume that that 1 mole of O_2_ produces one mole of CO_2_, as described by Eq 7.

% vol O_2_	Flow Rate (ml/min)	O_2_ Flow Rate (moles/min)	Max Mass of Carbon Removed (mg/min)
21	250	0.0021	25.77
21	100	0.0009	10.31
21	50	0.0004	5.15
21	25	0.0002	2.58
			
60	250	0.0061	73.61
60	100	0.0025	29.45
60	50	0.0012	14.72
60	25	0.0006	7.36

**Fig 10:**
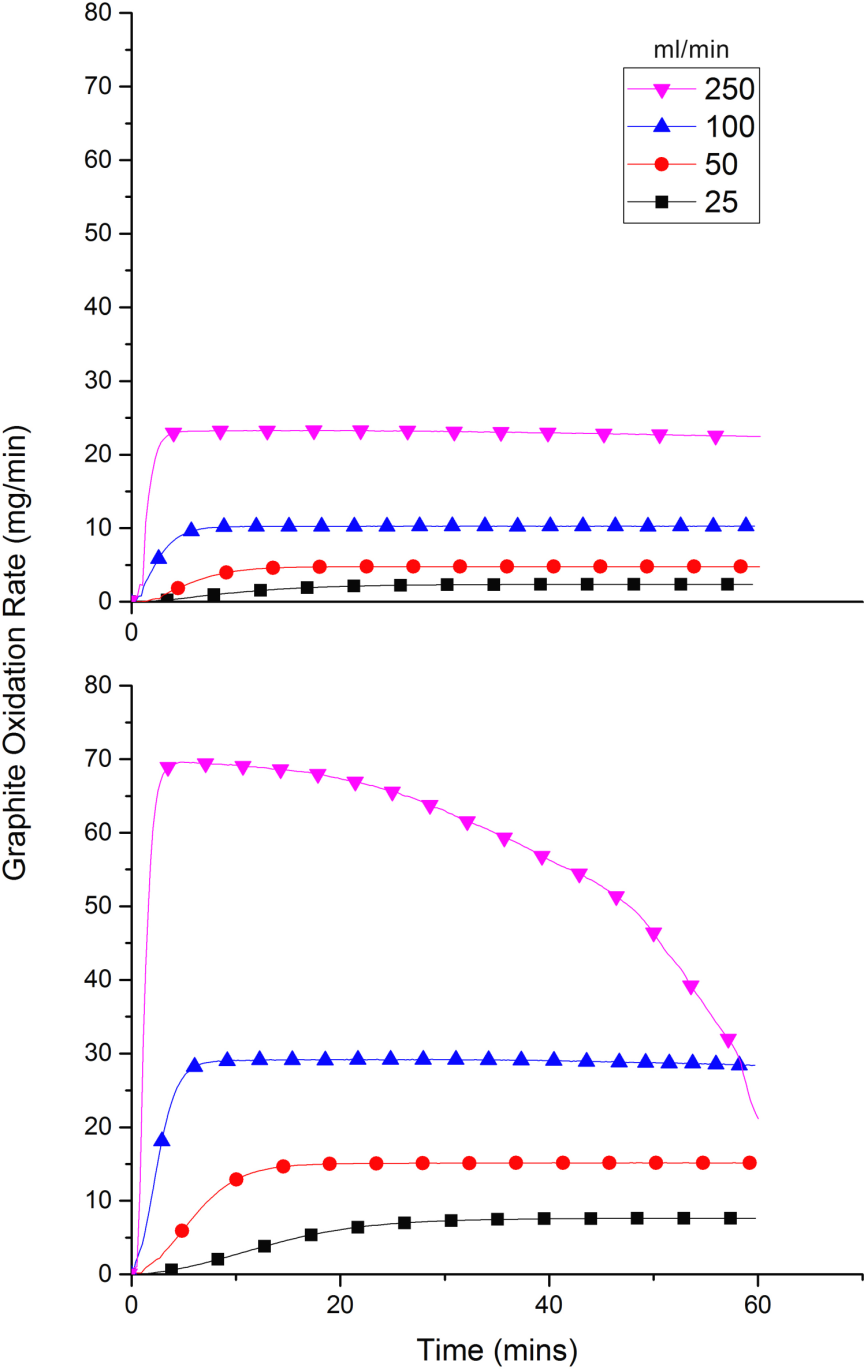
The effect of increasing oxidant flow rate on the graphite oxidation rate. Experiments carried out at 1000°C and in air (top) and 60% O_2_ (bottom).

Fig 10 shows the effect of increasing the flow rate of the oxidant on the oxidation rate in both air and 60% O_2_. The temperature was fixed at 1000°C and the flow rate was varied over the range 25–250 ml/min. In both instances the oxidation rate increases significantly with flow rate reaching a maximum of 23.3 and 69.6 mg/min, at a flow of 250 ml/min for air and 60% O_2_ respectively. In 60% O_2_, this maximum is reached rapidly and a steady-state is achieved, however, due to the rapid oxidation taking place via mode C the sample is quickly gasified and the overall mass diminishes rapidly. This leads to a corresponding drop in the oxidation rate as there is less sample left to oxidise. At the end of the experiment only 9.4 mg of sample remained, corresponding to a mass loss of 99.7%.

**Fig 11:**
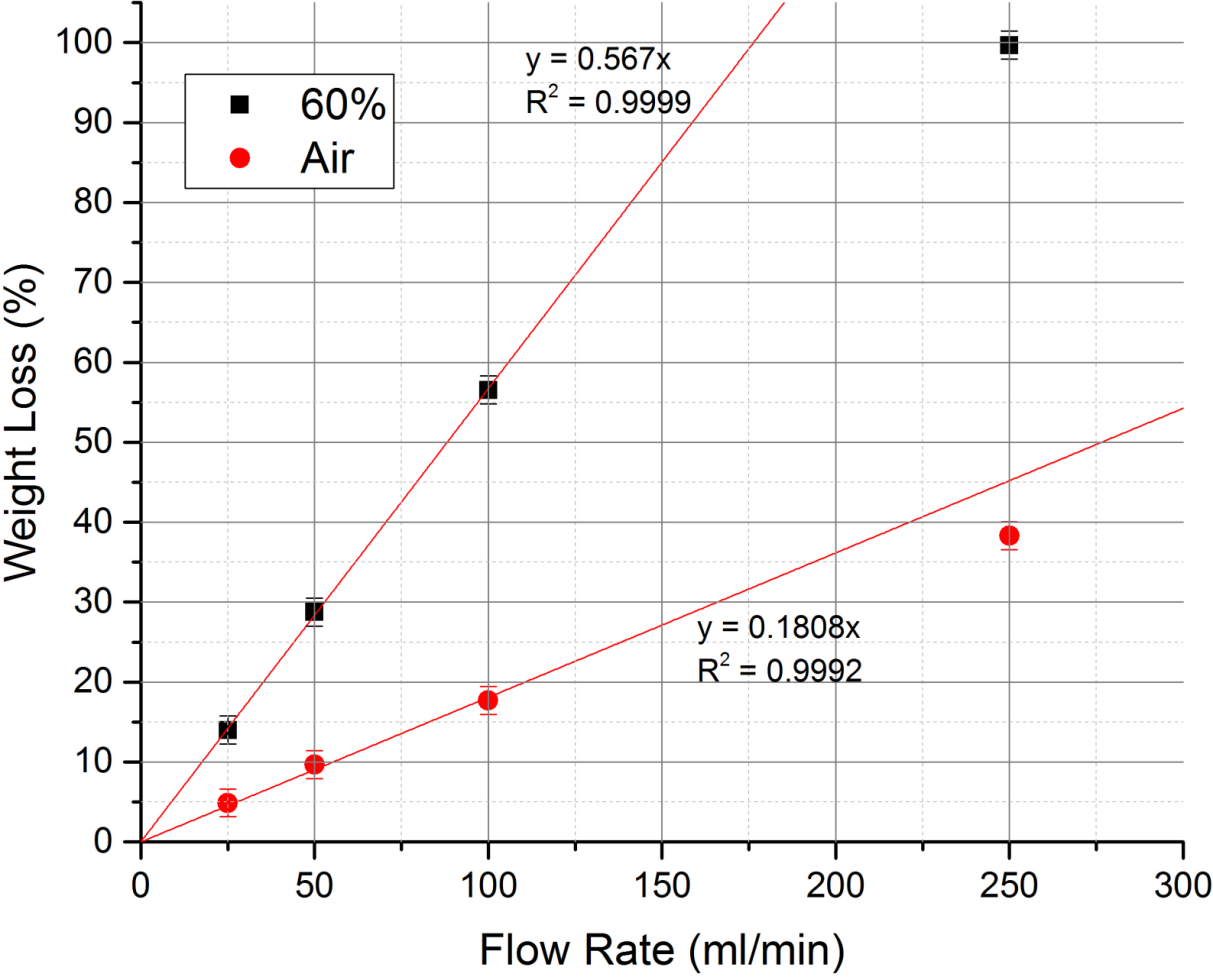
The effect of flow rate on oxidation induced weight loss at 1000°C in both air and 60% O_2_.

From Fig 11, the effect of flow rate on oxidation rate, at a fixed temperature, is found to be predominately linear, however, at high flow rates the trend starts to deviate from linearity suggesting that some ‘oxygen wastage’ is occurring, i.e. not all the oxygen is reacting with the graphite but instead passing straight through the system. This observation is particularly important for an industrial operation where any gas wastage would need to be avoided in order to maximise cost efficiency. From Fig 11 it can be seen that, for this particular experimental setup, an equivalent weight loss could have been achieved with flow rates of approximately 215 and 176 ml/min for air and 60% O_2_ respectively, and that the remainder of the 250 ml/min did not react.

A literature survey carried out by Contescu et al [55] concluded that increasing the flow rate actually changed the transition temperatures between the different kinetic regimes, and that the mechanism remained in mode A if the oxygen supply rate was approximately ten times higher than the rate of carbon oxidation. However, in this study, flow rates are comparatively limited and as such the oxygen supply is not enough to cause a similar effect. Also, in this study, graphite particulate is used as opposed to solid pieces described in the literature; this would lead to an enhanced surface area effect and a greater accessibility of active sites to accommodate any extra oxygen resulting from increased flow rates. The results collected here indicate a significant benefit from increasing flow rate on a pilot scale, although to remain commercially sound the amount of potential wastage should be reduced as much as possible by careful plant design.

## Conclusions

This study has shown that thermal treatment of nuclear grade graphite is an effective method of gasification to CO/CO_2_ and therefore a potentially viable graphite waste management option, at industrial scale. This work demonstrates that nuclear graphite particulate is readily oxidised in both air and 60% O_2_ and the oxidation rate, and therefore, the throughput, can be optimised through the control of several parameters which should be considered in the design of any potential treatment plant.

Primarily, the oxygen concentration will have the limiting effect on oxidation rate, with an almost linear relationship at suitably high temperature e.g., 1000°C. However, from a commercial point of view any improvement in oxidation rate would need to be weighed against the additional cost of large volumes of oxygen-rich gas. Despite the lower oxidation rates air may still be the oxidant of choice, at pilot-scale, due to the cost savings.

The flow rate of the oxidant also has a considerable effect, again exhibiting a linear relationship with oxidation rate at high temperature, although, it has been seen that this can deviate from linearity at high flow rates due to oxygen wastage. As a result this would need to be minimised from a commercial viewpoint and the design of the pilot scale plant would need to incorporate methods to avert this.

Oxidation rates can also be optimised by increasing the process temperature. At T ≤ 700°C, oxidation was found to proceed via the relatively slow diffusion-controlled mechanisms and at T ≤ 500°C the observed rates were considered negligible, hence such temperatures would not be suitable for an industrial scale process. A substantial increase in oxidation rate was observed at T > 800°C due to the transition from a diffusion–boundary layer controlled mechanism. Maximum oxidation rates were achieved at T > 1000°C, due to a rapid and significant increase in the CO/CO_2_ release ratio; this may be beneficial for a larger operation due to improved heat control arising from the CO production.
